# Consistent metagenes from cancer expression profiles yield agent specific predictors of chemotherapy response

**DOI:** 10.1186/1471-2105-12-310

**Published:** 2011-07-28

**Authors:** Qiyuan Li, Aron C Eklund, Nicolai J Birkbak, Christine Desmedt, Benjamin Haibe-Kains, Christos Sotiriou, W Fraser Symmans, Lajos Pusztai, Søren Brunak, Andrea L Richardson, Zoltan Szallasi

**Affiliations:** 1 Center for Biological Sequence Analysis, Department of Systems Biolology, Technical University of Denmark, 2800 Lyngby, Denmark; 2 Department of Medical Oncology, Dana-Farber Cancer Institute, Boston, MA 02115, USA; 3 Medical Oncology Department, Jules Bordet Institute, Brussels, 1000, Belgium; 4 Department of Biostatistics, Dana-Farber Cancer Institute, Boston, MA 02115, USA; 5 Department of Pathology, University of Texas M.D. Anderson Cancer Center, Houston, TX 77030, USA; 6 Department of Breast Medical Oncology, University of Texas M.D. Anderson Cancer Center, Houston, TX 77030, USA; 7 Department of Pathology, Brigham and Women's Hospital, Boston, MA 02115, USA; 8 Children's Hospital Informatics Program at the Harvard-MIT Division of Health Sciences and Technology (CHIP@HST), Harvard Medical School, Boston, MA 02115, USA

## Abstract

**Background:**

Genome scale expression profiling of human tumor samples is likely to yield improved cancer treatment decisions. However, identification of clinically predictive or prognostic classifiers can be challenging when a large number of genes are measured in a small number of tumors.

**Results:**

We describe an unsupervised method to extract robust, consistent metagenes from multiple analogous data sets. We applied this method to expression profiles from five "double negative breast cancer" (DNBC) (not expressing ESR1 or HER2) cohorts and derived four metagenes. We assessed these metagenes in four similar but independent cohorts and found strong associations between three of the metagenes and agent-specific response to neoadjuvant therapy. Furthermore, we applied the method to ovarian and early stage lung cancer, two tumor types that lack reliable predictors of outcome, and found that the metagenes yield predictors of survival for both.

**Conclusions:**

These results suggest that the use of multiple data sets to derive potential biomarkers can filter out data set-specific noise and can increase the efficiency in identifying clinically accurate biomarkers.

## Background

Microarray gene expression profiling provides an unbiased, comprehensive view of an entire molecular system, and is well suited to identify the relevant factors that define the cancer phenotype. However, the success of this method can be impeded by problems arising from the parallel measurements of tens of thousands of gene expression levels sampled in a far lower number of tumor specimens, typically a few hundred at most. Two specific problems have impacted cancer research: First, overfitting has produced several seemingly promising diagnostic patterns that have not been verifiable in independent studies [1,2]. Second, redundant information in the form of strongly correlated genes has led to the repeated "discovery" of diagnostic patterns detecting a single robust phenomenon, such as the cell proliferation pattern that is prognostic in estrogen receptor (ER) positive breast cancer [3]. One approach to these problems is to reduce the dimensionality of the data by combining (usually correlated) genes into a small number of metagenes.

Several gene combinations have been used to characterize the cancer phenotype [4-7]. For example, the linear combination of proliferation associated genes and estrogen regulated genes provides a better predictor of outcome in tamoxifen treated ER-positive breast cancer than does either class of genes alone [8]. Although several supervised methods to find biologically relevant linear gene combinations are available, finding such predictive metagenes in an unsupervised fashion remains a challenge [5,9]. In breast cancer, expression profiles can easily discriminate between ER-negative and ER-positive tumors, which have very different clinical behavior. For this reason it is also easy, but not clinically useful, to develop trivial predictors of outcome in cohorts of mixed ER subtype. Within the ER-positive subgroup, several predictors of response to chemotherapy have been described [10-12]. However, supervised methods have not yielded highly accurate predictors of chemotherapy response in DNBC [3,13,14]. This molecularly and clinically distinct subset of breast cancers represents approximately 20-25% of all breast cancers and can be treated only with chemotherapy. About 25-30% of these cancers respond favorably to treatment, but the remainder has very poor survival despite current best therapies [15].

Here we describe an unsupervised method to derive metagenes by leveraging the consistent expression patterns found in multiple gene expression data sets of the same cancer subtype. Our approach is based on the postulate that analogous microarray data sets, such as those from patient cohorts selected under similar criteria, are representative collections from a larger population "expression space". In this expression space, individual samples are robustly separated by a set of metagenes, some of which may be clinically relevant. However, each individual data set may be adulterated by sampling artifacts and with data set specific noise. Therefore, our approach is to derive metagenes that are consistently observed in several cohorts and are likely representative of the entire population. By first identifying metagenes in an unsupervised fashion, and then evaluating association between the metagenes and clinical outcome, we reduce the risk of overfitting.

Using this method we derived metagenes from expression profiles of DNBC, stage III ovarian cancer and early stage lung cancer, respectively. Then we verified the association of these metagenes with clinical outcome in independent validation cohorts of the three cancer types.

## Results

### Derivation of DNBC-specific consistent expression indices (CEIs)

We created a reference data set of DNBC from five previously published breast cancer cohorts that were all profiled on the same microarray platform (HG-U133A) and were without neoadjuvant drug response data [3,16-21] (Additional file 1). From a total of 1037 tumors we identified a subset of 218 DNBC based on expression levels of ESR1 and ERBB2 [3,4,22-24] (Additional file 2).

**Figure 1:**
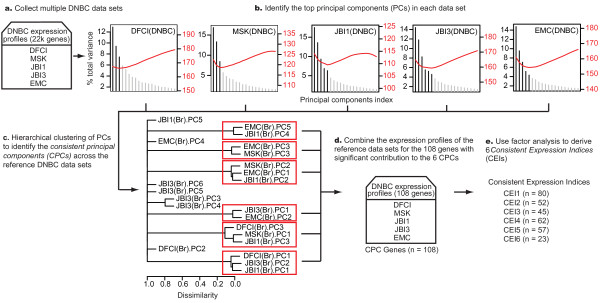
**Schematic of CPC analysis and CEI derivation, showing results from DNBC**.

First, we used principal component analysis (PCA) as an unsupervised method to identify a subset of genes representing highly variable patterns in DNBC expression profiles. In PCA, each principal component (PC) is defined by a vector of gene expression weights. We hypothesize that the between-sample variability of tumor is driven by a finite number of biological effects, which are summarized into the principal components. Hence a finite number of components will explain the majority of the variation of the data matrices. Therefore, we define the likelihood as the fraction of total variance that is explained by the given number of principal components. For each individual data set, we performed PCA and used the Bayesian information criterion (BIC) to select a set of 3-6 PCs that best represent the predominant variation in the data without including components that are likely to represent noise (Figure 1a, b; see methods). We expected to find any clinically relevant information enriched in these top PCs, since as the variance diminishes it becomes more difficult to distinguish signal from noise. For each reference data set, we distilled the PCs to include only the genes with a substantial contribution, as determined by the correlation between gene expression levels and PC scores across all samples. Hierarchical clustering of these distilled PCs revealed six distinct groups, or *consistent principal components *(CPCs), with at least two members. We identified 108 genes with a substantial contribution to at least two PCs in any of these clusters, hypothesizing that these genes are likely to capture consistent biologically-relevant information about DNBC (*CPC genes*) (Figure 1c).

**Figure 2:**
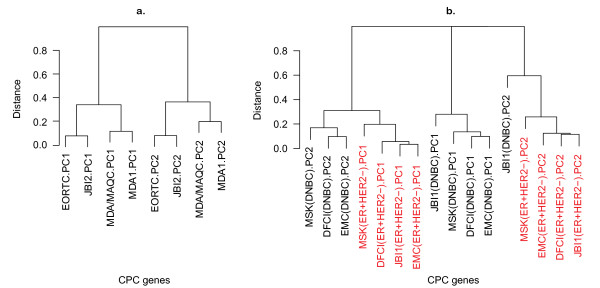
**The CPC genes yield consistent, subtype-specific PCs in gene expression data sets**. In each panel, PCA was performed separately on each data set using only the CPC genes, and the resulting first and second PCs from each data set were compared by hierarchical clustering. (**a**) The first two PCs of the 108 CPC genes in the DNBC subset of four validation data sets. (**b**) The first two PCs of the 108 CPC genes in the DNBC subset (black) and the ER-positive HER2-negative subset (red) of the four validation data sets.

To validate the consistency of these CPC genes, we collected four independent DNBC data sets and subjected them to PCA using only the 108 CPC genes [13,25-27]. As result, the first and the second principal components of the CPC genes are highly consistent across the four test data sets, suggesting that these genes correspond to conserved biological variation in DNBC (Figure 2a). When we applied this gene set to the ER-positive HER2-negative subset of the same cohorts, we found that the resulting top PCs were distinct from those of the DNBC samples (Figure 2b). Thus, the CPC genes represent a specific type of variation of gene-expression within DNBC, which is highly conserved in multiple different cohorts.

Next we used factor analysis (FA) to distill the information in the CPC genes into six biologically relevant metagenes (Figure 1d, e). FA can be considered an extension of PCA in which an additional rotation maximizes variance of the gene weights. This additional rotation step results in a more even distribution of variance among components than does PCA alone. In general, FA is often preferred when the goal of the analysis is to understand and explain the structure in the data [28]. Using only the CPC genes in the combined reference data sets, we identified six factors that together explained 57% of the variance in the CPC genes (Additional file 3). In order to estimate the contribution of these factors in other data sets, we defined six *consistent expression indices *(CEIs) based on the sign of the non-trivial gene weights from each factor; thus each CEI comprises between 23 and 80 of the CPC genes (Additional file 3). At this point the CEIs were finalized, and in all subsequent analysis the CEIs were applied to the data sets without further adjustment. Thus, the CEIs were derived entirely from expression data, without consideration of any functional annotation or clinical outcome.

### Association between CEIs and clinical outcome in double-negative breast cancer

We hypothesized that the six CEIs, which account for highly conserved biological variation among DNBC cases in the five reference data sets, are also associated with certain clinical phenotypes of the tumors. We investigated whether the CEIs were predictive of response to specific treatment regimens in four independent test cohorts in which expression profiles were obtained from DNBC samples prior to neoadjuvant therapy (Table 1). Two of these cohorts, MDA1 [26] and MDA/MAQC [13], were similar: the samples were acquired by fine needle aspiration, and the patients received paclitaxel, fluorouracil, doxorubicin, and cyclophosphamide (TFAC). In contrast, the two other data sets were derived from core biopsies; one cohort, EORTC, received fluorouracil, epirubicin and cyclophosphamide (FEC) [25], whereas the other cohort, JBI2, received only epirubicin [27] (Table 1).

**Table 1:** DNBC-derived CEIs are associated with tumor response to neoadjuvant chemotherapy in DNBC cohorts

				AUC					
cohort	regimen	patients	responders	CEI1	CEI2	CEI3	CEI4	CEI5	CEI6
EORTC	FEC	37	16	0.73^R^*	0.57	0.51^R^	0.61	0.56	0.54
MDA1	TFAC	27	13	0.78 **	0.62	0.77**	0.61^R^	0.53	0.61
MDA/MAQC	TFAC	30	9	0.77*	0.66	0.78*	0.62^R^	0.58	0.54
DFCI2	P	24	4	0.73	0.72^R^	0.50	0.52^R^	0.52^R^	0.57^R^
JBI2	E	43	4	0.85^R^*	0.73^R^	0.53	0.88**	0.58^R^	0.72

Each CEI was evaluated as a univariate predictor of pathological complete response or residual disease using the area under the ROC curve (AUC). Chemotherapy regimens are indicated: A, doxorubicin; C, cyclophosphamide; E, epirubicin; F, 5-fluorouracil; P, either cisplatin or carboplatin; T, either paclitaxel or docetaxel. The CEIs were derived from four independent DNBC cohorts not shown in this table. * *P *< 0.05; ** *P *< 0.01. R: AUC is estimated based on association to residual disease (RD).

**Figure 3:**
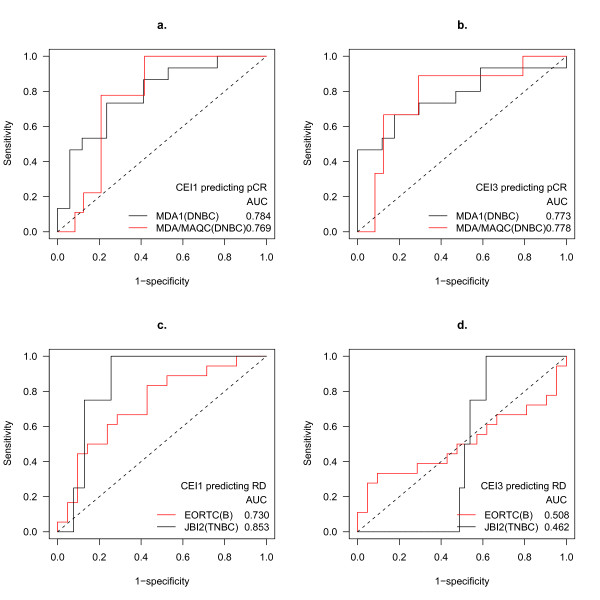
**High CEI1 and CEI3 scores are associated with agent-specific response to neoadjuvant therapy**. DNBC patients were given neoadjuvant TFAC (MDA1, MDA/MAQC), FEC (EORTC) or epirubicin only (JBI2). ROC curves indicate the association between (**a**) high CEI1 or (**b**) high CEI3 and pathological complete response (pCR) to taxane-based chemotherapy; and (**c**) high CEI1 or (**d**) high CEI3 and non-pCR to non-taxane-based chemotherapy.

We evaluated the association between pathologic complete response (pCR) and each of the six CEIs using area under the receiver operating characteristic (ROC) curves (AUC). In the MDA1 data set we observed a strong positive association between CEI1, CEI3 and pCR (AUC = 0.78, *P *= 0.005 for CEI1, AUC = 0.77, *P *= 0.009 for CEI3, Table 1). Similar associations were also observed in the second TFAC data set, MDA/MAQC (AUC = 0.77, *P *= 0.02 for CEI1, AUC = 0.78, *P *= 0.001 for CEI3, Table 1, Figure 3a, b).

In the two cohorts in which patients received neoadjuvant chemotherapy without taxane, we found CEI1 is significantly associated with residual disease (RD), a typical poor pathological response (AUC = 0.73, *P *= 0.01 in EORTC, AUC = 0.85, *P *= 0.02 in JBI2). On the other hand, there is no detectable association between CEI3 and response to either FEC or epirubicin treatment (Table 1, Figure 3c, d). These associations between CEIs and pathological responses in the validation cohorts was stronger than any we observed using published predictors [25,26] or using predictors we derived using conventional methods (Additional file 4).

**Figure 4:**
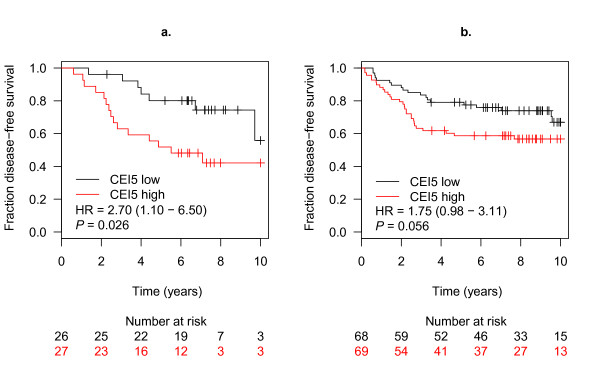
**CEI5 is associated with outcome in DNBC patients who received adjuvant chemotherapy but not in patients who received no adjuvant chemotherapy**. The EMC, JBI1, GIS, KUH, UCSF and NKI cohorts were combined, and patients were grouped according to subtype and presence of adjuvant therapy. Within each group, patients were stratified according to median of CEI scores, and disease-free survival was compared. (**a**) CEI5 in adjuvant treated tumors; (**b**) CEI5 in tumors without adjuvant therapy.

Since pathological response to chemotherapy is based only on short-term follow-up, we also examined the association of these CEIs and long-term clinical outcome after chemotherapy. In a pooled DNBC cohort of 236 patients for which follow-up data is available (Additional file 1), of all the six CEIs, we found that binary classification based on CEI5 was significantly associated with disease-free survival of patients who received adjuvant chemotherapy within 10 years of follow-up (*HR *= 2.70, *P *= 0.026, Figure 4).

To test whether the CEIs were simply capturing known metagenes, we compared the six CEIs with 38 signatures reflecting tumor-associated biological processes or infiltrating cell types [25]. We used a meta-analysis based on seven data sets and found CEI1 was negatively correlated with ER/luminal-basal metagenes and ERBB2-molecular apocrine tumor metagenes; whereas CEI3 was positively correlated with the proliferation/AURKA metagene (Additional file 5). We also observed other correlations: CEI3 negatively correlated with the stroma and adipocyte metagenes. However, none of these metagenes was reported to hold similarly strong and consistent predictive power in the original studies as that of CEI1 and CEI3 [25] (Additional file 4). This may suggest that synergistic effects of multiple biological processes are more deterministic of the response to therapy than any single ones. In addition, CEI5 and CEI6 were not correlated with any of the known metagenes. Therefore, these two CEIs may reflect some biological processes relevant to DNBC but not yet described as such in any previous study.

### Comparison with existing methods

In order to compare the performance of the CPC approach to existing algorithms, we assessed several supervised and unsupervised methods for their ability to generate metagenes predictive of treatment response.

For supervised methods, we first selected genes that are significantly associated with pathological response to taxane-based neoadjuvant therapy in the MDA1 data set based on Pearson's correlation coefficients, diagonal linear discrimination analysis [26,29], student's t-test, Wilcoxon's rank sum test, or nearest shrunken centroids [30]. We validated the predictive power of these metagenes in two other cohorts, MDA2 and EORTC. Metagenes based on Pearson correlation coefficients and nearest shrunken centroids yielded consistently significant predictions in the test data sets whereas the rest of the methods did not (Additional file 4). However, the predictive power represented by the area under the curves (AUCs) of all gene-by-gene methods decrease in the validation cohorts, suggesting overfitting..

For unsupervised methods, we pooled the five DNBC data sets and subjected it to independent component analysis (ICA) [31] or sparse principal component analysis (SPCA) [32]. Three of the six top ICA components were predictive of pathological response in MDA1 and MDA2 data sets; and three of the six top SPCA components were predictive of pathological response in MDA1 and JBI2 data sets; whereas with the same number of components, consistent expression indices were predictive in four cohorts. More importantly, these methods produced less consistent results in terms of their predictive power in the two cohorts with similar treatment regimen. None of the components derived by ICA and SPCA, predicted the pathological response in the two taxane-based neoadjuvant trials (MDA1 and MDA/MAQC) in a consistent fashion. In particular, the third and fifth independent components (ICA3 and ICA5) predicted outcome the opposite direction, high values predicting favorable response in one and unfavorable response in the other cohort (Additional file 6).

### Other cancer types

#### ER-positive HER2-negative breast cancer

The ER-positive HER2-negative tumor is another major subtype of breast cancer and differs from DNBC in both transcriptional and genomic features [4]. Since some of the DNBC-derived CEIs may capture consistent biological variations common to both subtypes, we examined the association between the DNBC-derived CEIs and clinical outcome in ER-positive HER2-negative subsets of the validation cohorts. In a pooled cohort of 858 ER-positive HER2-negative tumors [9,21,33-36], binary classification based on CEI3 was significantly associated with disease-free survival in tamoxifen-treated patients (*HR *= 3.20, *P *= 0.016) as well as in patients not given tamoxifen treatment (*HR *= 1.8, *P *= 0.0004) (Additional file 7). Compared to DNBC, where CEI3 was associated with only pathological response to TFAC therapy but not long-term clinical outcome, the prognostic power of CEI3 in ER-positive HER2-negative tumors suggests that the same biological process, proliferation, may have different effects in the two different subtypes, which is concordant with previous translational studies performed in ER-positive tumors [3,37,38].

#### Ovarian cancer

Ovarian cancer is represented in only a limited number of microarray data sets and to the best of our knowledge there are no two analogous ovarian cancer data sets for which the same type of clinical outcome data is publicly available. Therefore, this type of cancer offered an opportunity to test our proposition that clinically relevant predictors can be extracted from data sets not associated with (and trained on) clinical outcome data.

**Figure 5:**
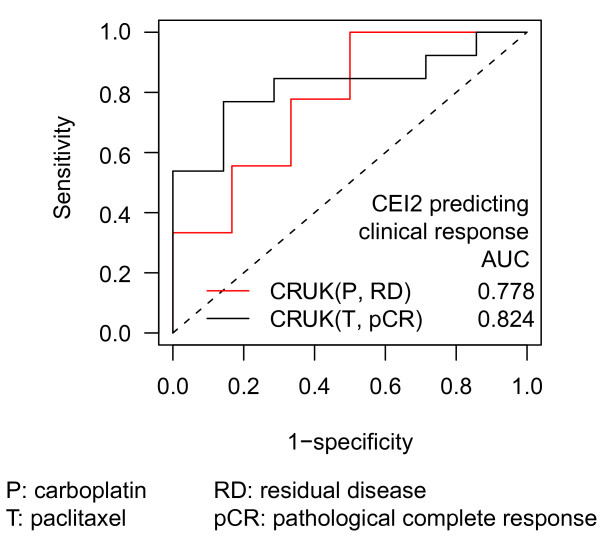
**Consistent expression indices derived from stage III ovarian cancer are associated with treatment response**. Ovarian cancer-derived CEI2 predicted pathological complete response (pCR) to paclitaxel monotherapy and non-pCR to carboplatin monotherapy in CRUK ovarian cancer cohort.

We tested whether the CEIs derived from three stage III ovarian cancer data sets, EXPO‡, AOC and DU [39-41], predict treatment response or clinical outcome in other independent ovarian cancer cohorts (Additional file 3). In the BIDMC cohort [42], CEI1 derived from ovarian cancer was significantly associated with overall survival in 5 years after chemotherapy (HR = 8.36, *P *= 0.011, Additional file 8). Additionally, in the CRUK cohort [43], in which patients were assigned randomly to two groups treated with either paclitaxel or carboplatin monotherapy, CEI2 was associated with good response (pCR) to paclitaxel (AUC = 0.82, *P *= 0.02) but with poor response (RD) to carboplatin (AUC = 0.78, *P *= 0.09, Figure 5).

#### Lung adenocarcinoma

Finally, we turned our attention to lung adenocarcinoma, for which at least five microarray data sets are publicly available [39,44]. In a recent multi-site blinded validation study, at least eight gene expression based survival predictors were tested in two validation data sets, but none of these predicted clinical outcome in stage I cases in more than one data set unless clinical covariates were included [44]. Therefore, we applied the same strategy to early stage lung cancer. In order to test our method within the same analytical framework of the original study we applied a cross-validation approach in the four lung cancer cohorts by extracting CEIs from each combination of three cohorts (using early stage samples only) and testing for association between these lung cancer-derived CEIs and outcome in the remaining cohort (for stage I only). In three of the four rounds of the validation, at least one of the CEIs were significantly predictive of outcome in stage I lung cancer in the validation cohort, without the use of further clinical variables and without any training on outcome (Additional file 8). Furthermore, we derived four CEIs from all four lung cancer data sets (early stage only, Additional file 3) and tested them on a fifth independent lung cancer cohort [39] and found that CEI1 was predictive of 5-year overall survival in stage I samples (HR = 7.73, *P *= 0.034, Additional file 8).

To understand the biology underlying the predictive power of these CEIs, we tested for enrichment of Gene Ontology (GO) annotations for biological processes in the CPC genes. For the CPC genes of the DNBC derived CEIs, the most enriched GO categories included immune and inflammatory response. For the lung cancer derived CEIs, the top categories included digestion, response to external stimulus, and oxidation/reduction (Additional file 9). While the GO category analysis did not provide an easy interpretation of the observed predictive power of clinical behavior, a literature analysis identified several genes that were linked to specific chemotherapy response or resistance mechanisms, including GPX3 [45], HPGD [46], AKR1C1, and AKR1C2 [47].

## Discussion

We have presented a method to extract metagenes that consistently distinguish among individual double-negative breast cancers in multiple gene expression data sets. We found a strong association between three of the six CEIs and the efficacy of various neoadjuvant treatments in DNBC. This association was stronger than that of previously published predictors and suggests that these gene sets reflect important biological processes that influence sensitivity to chemotherapy. Importantly, different CEIs were predictive of different regimens. Furthermore, some CEIs were predictive only in DNBC and not in ER-positive tumors.

An attractive feature of the method presented here is that it is unsupervised; i.e. the CEIs are derived without information about clinical response or outcome. This holds particular importance for cancer types with only a few existing clinical outcome matched microarray based cohorts [48]. In the case of cancer types of higher incidence and easier access to clinical material (e.g. breast, lung), multiple analogous cohorts complete with clinical outcome data, often up to six or seven independent data sets, are available for supervised analysis to identify individually informative genes. These genes could then be combined into multi-gene prediction models and independently validated on the various cohorts. In the case of other cancer types (pancreas, prostate, etc.), lower incidence, difficulties with obtaining appropriate RNA material, or the specific clinical course of the disease results in a lack of clinical outcome matched microarray data sets. In such cases a method that is able to extract potential outcome predictors without training on outcome data may provide a potential solution. Given the observation that CEIs may already hold predictive value without being fitted to the actual clinical outcome, CPC-based methods may extract testable predictors from microarray data without matched clinical outcome, and the few outcome matched microarray cohorts could then be used for independent validation. For example, prostate cancer is represented by at least fourteen microarray cohorts, but only three of these have clinical outcome published as well [49-52].

Although biological functions of the CEIs can be partially understood by methods such as GO analysis, our knowledge about these genes still remains very limited. There might be several reasons for this. First, many of the genes listed in the CEIs have not been investigated in detail for direct involvement in drug resistance mechanisms. Second, drug resistance might be the result of a distinct but complex biological feature which involves a concert of relevant biological mechanisms, such as increased expression of multidrug resistance genes, low proliferation rate, and the combination of these mechanisms might be best quantified by common upstream and downstream markers that reflect the expression level the relevant biological mechanisms. In general, it is desirable for clinical predictors to be associated with uniquely identifiable biological mechanisms so as for therapeutic targetability. However, we emphasize that our approach was designed to overcome the failure of single gene, single biological mechanism prediction of clinical outcome [53]. We aimed at determining and testing the utility of the most robust and consistent information in high throughput data sets, which is more likely to capture the most comprehensive and dominant biological variations in human tumors rather than any single unique biological process from limited prior knowledge.

The predictors presented in this paper would need to be refined before introduction into clinical practice. Currently each CEI comprises up to 235 genes, a number that might be impractical for a clinical test such as multiple quantitative PCR. Also, treatment decisions are dichotomous; a patient either receives a particular treatment or does not. Therefore, the most useful clinical tests have decision thresholds, which will need to be determined for the CEIs and will need to be validated in independent cohorts to establish the sensitivity and specificity of a future treatment response test.

## Conclusion

The approach we described in this analysis is well-suited to identify linear gene combinations that express consistent variations in a set of independent but biologically similar datasets, regardless of the observed clinical outcome. The ability of these metagenes to predict response to chemotherapy has been evaluated in completely independent set of cohorts. Unlike other existing unsupervised methods, by mandating the consistency of the weights of genes in the loading matrix, the consistent principal components are more likely to yield reproducible predictive power.

## Methods

### Data sets

All microarray data sets used in this study were previously published and are available from several public data repositories, except for the BIDMC ovarian cancer data set, which was obtained from the authors [42]. Each microarray data set was processed with RMA [54]. For each cohort, a list of samples used in the analysis is provided in Additional file 1.

To determine the double-negative breast cancer (DNBC, not expressing ESR1 or HER2), we clustered each data set based on the probe levels of ESR1 and HER2 using the Partitioning Around Medoids (PAM) algorithm. The DNBC is determined by the cluster with consistent low expression of both genes.

### Consistent Principal Components Analysis

For each of the reference data sets independently, we computed the coefficient of variation (CV) based on the anti-logarithm of RMA probe levels and kept probe sets with a CV greater than one and less than 1000; thus we selected 614 to 1714 probe sets from each data set. Next we performed PCA on these highly variable probe sets in each data set, and selected an optimal number *k *of top PCs by the minimum of the BIC:

Here, *n *is the number of samples, *k *is the number of components selected, and *ν *is the unexplained variance which equals the residual sum of squares, given by:

Here, *σ*_*i *_is the standard deviation of probe set *i, p *is the number of probe sets, and *ω*_*j *_is the standard deviation explained by PC *j *(equal to the square root of the *j*'th eigenvalue). For each PC, we calculated the Pearson correlation coefficient (PCC) between its component scores and the expression level of each probe set and the significance of the correlation is assessed by Student's t-test. Probe sets with a *P *< 0.01 for PCC were selected to represent the PC. After the selection, each PC contains 42 to 211 representative probe sets.

To compare PCs derived from various data sets, we defined the following measure of the dissimilarity between PCs *i *and *j*:

Where *J*_*ij *_is the Jaccard index (the ratio between size of the intersection and the size of the union of the representative probe sets of component *i *and *j*) and *C*_*ij *_is the cosine correlation coefficient between the weights of the common representative probe sets of component *i *and *j*.

We used this distance function to perform average linkage hierarchical clustering on the selected PCs from all reference data sets. For each distinct cluster, we selected the set of genes found in at least two members.

### Factor analysis and CEI calculation

We retrieved the RMA expression profile of the CPC genes from the reference data sets. When a gene was represented by multiple probe sets, we selected the probe set with largest standard deviation to represent that gene. For each of the expression matrices retrieved, we computed the standard z-scores for each gene and merged the matrices into one.

We performed factor analysis of the merged z-scores using the "varimax" rotation and with the number of factors set to six [28]. For each factor we estimated the gene coefficients using the least-square method. Coefficients with an absolute value below 0.1 were set to zero, and the signs of the coefficients were used as the gene weights in the corresponding CEI.

### Prediction and prognosis

The ROC curves were based on individual CEI scores and treatment response. We calculated the area under the curve (AUC) using the trapezoidal rule [55] and estimated statistical significance using the Wilcoxon rank sum test. Survival curves were generated using the Kaplan-Meier method. Hazard ratios were estimated for 5 year or 10 year follow-up by Cox regression in which the patients were stratified into two groups of equal size according to the median of the CEI score. Statistical significance was estimated using the log rank test.

Further details are available in Additional file 2.

## Competing interests

The authors declare that they have no competing interests.

## Authors' contributions

QL conceived the study, analyzed the data and helped draft the manuscript; ACE, NJB participated in the data analysis and helped draft the manuscript; CD, BH and CS contributed data and participated in the data analysis; WFS, LP contributed data; SB helped draft the manuscript; ALR contributed data and helped draft the manuscript; ZS conceived the study and drafted the manuscript. All authors read and approved the final manuscript.

## Supplementary Material

Additional file 1 **Summary of the tumor expression data sets used in this study**. **(a) **Summary of all data sets used in this manuscript; **(b) **The number of DNBC samples from each data set used in each figure; **(c) **The number of ER-positive/Her2-negative breast cancer samples from each data set used in each figure; **(d) **The number of ovarian cancer samples from each data set used in each figure; **(e) **The number of lung cancer samples from each data set used in each figure. Click here for file

Additional file 2 **Supplementary methods**. Supplementary methods. Click here for file

Additional file 3 **CEIs derived from three tumor types**. CEIs derived from DNBC, Stage III ovarian cancer and early-stage lung cancer by consistent principal component analysis. Click here for file

Additional file 4 **AUCs and P values for prediction of TFAC response**. Summary of AUCs and P values for prediction of TFAC response in DNBC using published metagenes and signatures derived using various supervised methods. Click here for file

Additional file 5 **Correlation between DNBC-derived CEIs and known metagenes**. Colorgram showing the pooled Pearson correlation coefficients between DNBC-derived CEIs and known metagenes. Click here for file

Additional file 6 **AUCs for prediction of pathological response in five DNBC cohorts which received neoadjuvant chemotherapy of different regimens using various unsupervised methods**. (a) CEIs derived from consistent principal components; (b) Components derived using independent component analysis; (c) Components derived using sparse principal component analysis. The pooled correlation coefficients were estimated from seven breast cancer data sets based on a meta-analysis. Click here for file

Additional file 7 **DNBC-derived CEI3 predict clinical outcome of in ER-positive HER2-negative breast cancer**. (a) ER-positive HER2-negative samples which received endocrine or radio-therapy from the EMC, JBI1, GIS, KUH, UCSF and NKI cohorts; (b) ER-positive HER2-negative samples which received no systematic therapy. Click here for file

Additional file 8 **Validation of the association between CEIs and clinical outcomes in ovarian cancers and lung cancers**. **(a) **Hazard ratios based on 5-year follow-up of three ovarian cancer-derived CEIs in the validation cohort (DU) based on univariate and multivariate Cox regression; **(b) **Summary cross-validation of CEIs derived from three early-stage lung cancer data sets and validated in the fourth for the association to clinical outcomes; **(c) **hazard ratios based on 5-year follow-up of seven lung cancer-derived CEIs in the validation cohort (DU) based on univariate and multivariate Cox regression. Click here for file

Additional file 9 **Gene Ontology (GO) annotation analysis**. Gene Ontology of CEIs derived from **(a) **DNBC, from **(b) **stage III ovarian cancer, and from **(c) **early stage lung cancer. Click here for file
