# Supplementary material for: Consistent metagenes from cancer expression profiles yield agent specific predictors of chemotherapy response
Source: BMC Bioinformatics. 2011 Jul 28;12:310. doi: 10.1186/1471-2105-12-310 (PMC3155975; doi:10.1186/1471-2105-12-310)
Supplement: Additional file 1 — Summary of the tumor expression data sets used in this study. (a) Summary of all data sets used in this manuscript; (b) The number of DNBC samples from each data set used in each figure; (c) The number of ER-positive/Her2-negative breast cancer samples from each data set used in each figure; (d) The number of ovarian cancer samples from each data set used in each figure; (e) The number of lung cancer samples from each data set used in each figure. [file 1471-2105-12-310-S1.DOC]

**Supplementary Table 1.** Summary of the tumor expression data sets used in this study.

**(a)** Summary of all data sets used in this manuscript.

| **Cohort** | **Tissue** | **Cohort size** | **Reference** | **Source** |
| --- | --- | --- | --- | --- |
| DFCI | Breast | 127 | 20-22 | GEO: GSE5460 |
| EMC | Breast | 286 | 23 | GEO: GSE2034 |
| JBI1 | Breast | 327 | 8, 19 | GEO: GSE2990 |
| JBI3 | Breast | 198 | 2 | GEO: GSE7390 |
| MSK | Breast | 99 | 18 | caArray Experiment:1015897589711211:1 |
| GIS | Breast | 289 | 32 | GEO: GSE4922 |
| KUH | Breast | 159 | 33 | GEO: GSE1456 |
| UCSF | Breast | 130 | 31 | caArray:Experiment:1015897589973255:1 |
| NKI | Breast | 295 | 4, 34 | http://www.rii.com/publications/2002/nejm.html |
| EORTC | Breast | 102 | 27 | GEO: GSE4779 |
| JBI2 | Breast | 120 | 19 | GEO: GSE16446 |
| MDA1 | Breast | 133 | 28 | http://bioinformatics.mdanderson.org/pubdata.html |
| MDA/MAQC | Breast | 100 | 12 | GEO: GSE16716 |
| DU | Ovary | 146 | 37 | http://data.cgt.duke.edu/oncogene.php |
| AOC | Ovary | 285 | 38 | GEO: GSE9891 |
| EXPO | Ovary | 204 | 39 | GEO: GSE2109 |
| BIDMC | Ovary | 65 | 15 | obtained from the authors |
| CRUK | Ovary | 35 | 40 | GEO:GSE15622 |
| CAN/DF | Lung | 88 | 41 | caArray:Experiment:1015897590633482:1 |
| HLM | Lung | 105 | 41 | caArray:Experiment:1015897590633482:1 |
| UM | Lung | 204 | 41 | caArray:Experiment:1015897590633482:1 |
| MSK | Lung | 107 | 41 | caArray:Experiment:1015897590633482:1 |
| DU | Lung | 111 | 37 | http://data.cgt.duke.edu/oncogene.php |

Note: "caArray" data can be downloaded here: https://caarraydb.nci.nih.gov/caarray/publicSearch.do?mode=experiments

**(b)** The number of DNBC samples from each data set used in each figure.

| **Cohort** | **Tissue** | **Derivation of DNBC CEIs** | **Fig. 1*** | **Fig. 2a (black)** | **Fig. 3,**  **Table 1** | **Fig.**  **4a** | **Fig.**  **4b** | **Supp.**  **Fig. 1** |
| --- | --- | --- | --- | --- | --- | --- | --- | --- |
| DFCI | DNBC | Y | 40 | - | - | - | - | 40 |
| EMC | DNBC | Y | 64 | - | - | - | 64 | 64 |
| JBI1 | DNBC | Y | 32 | - | - | - | 23 | 32 |
| JBI3 | DNBC | Y | 46 |  |  |  |  | 46 |
| MSK | DNBC | Y | 36 | - | - | - | - | 36 |
| GIS | DNBC | N | - | - | - | - | 21 | - |
| KUH | DNBC | N | - | - | - | 27 | - | - |
| UCSF | DNBC | N | - | - | - | 7 | 1 | - |
| NKI | DNBC | N | - | - | - | 16 | 28 | - |
| EORTC** | basal | N | - | 39 | 39 | - | - | 39 |
| JBI2*** | DNBC | N | - | 120 | 43 | - | - | 120 |
| MDA1 | DNBC | N | - | 32 | 32 | - | - | 32 |
| MDA/MAQC | DNBC | N | - | 38 | 38 | - | - | 38 |

* Derivation of DNBC-specific CPC genes and CEIs

** For the EORTC cohort, we used basal subtype as determined by Farmer et al.

*** Although only 43 JBI2 samples were defined as double-negative, we used the entire cohort of 120 samples for Fig. 2a, b and Supp. Fig. 1.

**(c)** The number of ER-positive / Her2-negative breast cancer samples from each data set used in each figure.

|  |  | **Fig. 2b** | **Supp. Fig. 2a** | **Supp. Fig. 2b** |
| --- | --- | --- | --- | --- |
| **Cohort** | **Tissue** | **(red)** | **(with tamoxifen)** | **(without tamoxifen)** |
| DFCI | Breast ER+/Her2- | 73 | - | - |
| EMC | Breast ER+/Her2- | 191 | - | 175 |
| JBI1 | Breast ER+/Her2- | 235 | 57 | 85 |
| MSK | Breast ER+/Her2- | 51 | - | - |
| GIS | Breast ER+/Her2- | - | - | 108 |
| UCSF | Breast ER+/Her2- | - | 26 | 2 |
| NKI | Breast ER+/Her2- | - | 14 | 102 |

**(d)** The number of ovarian cancer samples from each data set used in each figure.

| **Cohort** | **Tissue** | **Derivation*** | **Fig. 5a,b** |
| --- | --- | --- | --- |
| AOC | Ovary | 184 |  |
| EXPO | Ovary | 62 |  |
| DUKE | Ovary | 104 |  |
| BIDMC | Ovary |  | 57 |
| CRUK | Ovary |  | 35 |

* Derivation of ovarian cancer-specific CPC genes and CEIs

**(e)** The number of lung cancer samples from each data set used in each figure.

|  |  |  | **Fig.6** | **Supp Fig. 3** |
| --- | --- | --- | --- | --- |
| **Cohort** | **Tissue** | **Sample used to derive lung CEIs** | **(stage I only)** | **(stage I only)** |
| CAN/DF | Lung, early stage | 71 | - | 56 |
| HLM | Lung, early stage | 53 | - | 41 |
| UM | Lung, early stage | 77 | - | 116 |
| MSK | Lung, early stage | 56 | - | 63 |
| DU | Lung, early stage | 0 | 41 | - |
