# Supplementary material for: Consistent metagenes from cancer expression profiles yield agent specific predictors of chemotherapy response
Source: BMC Bioinformatics. 2011 Jul 28;12:310. doi: 10.1186/1471-2105-12-310 (PMC3155975; doi:10.1186/1471-2105-12-310)
Supplement: Additional file 2 — Supplementary methods. Supplementary methods. [file 1471-2105-12-310-S2.DOC]

## Supplementary Methods

### Identification of double-negative samples

Raw data (CEL files) for the "reference" data sets (EMC(1), MSK(2), JBI1(3-4), JBI3 and DFCI(5-7)) were processed separately by robust multi-array average (RMA)(8). For each data set, we selected "double negative" tumors: those that were negative for estrogen receptor (ER) and epidermal growth factor receptor 2 (HER2) based on clustering of samples with consistent low level of ESR1 and ERBB2 expression using the Partitioning Around Medoids (PAM) algorithm(9). T

### Identification of principal components from each data set

The following steps were performed on each of the five data sets independently. Except where noted in the "primary filtering" section, all analysis was performed on the "log_2_" expression levels, as produced by the RMA algorithm.

#### Primary filtering of probe sets

To focus on the subset of genes with the most substantial variation in expression, we performed a primary filtering of probe sets based on the coefficient of variation (CV, the ratio between standard deviation and mean) of expression level (here given by the base-2 anti-logarithm of RMA expression level). We considered a probe set sufficiently variable if the corresponding CV was larger than one. In order to leave out probe sets with extraordinarily high variance, we also set an upper limit of 1000. Thus, only probe sets with a CV ranges from 1 to 1000 were kept for further analysis (10). Thus we selected 614 to 1714 probe sets from each data set.

#### PCA and selection of components

After filtering, principal components analysis (PCA) was performed for each data set separately. The components were sorted in descending order by the fraction of total variance they explain. We used the Bayesian information criterion (BIC) to identify the optimal number of components.

$$\text{BIC}=n\ln\left(\frac{\nu}{n}\right)+k\ln(n)$$

Where *n* is the number of samples, and *k* is the number of components selected. The unexplained variance, *ν*, is given by the difference between total variance and sum of variance explained by the first *k* components.

$$\nu=\sum_{i=1}^{p} \sigma_{i}^{2}-\sum_{j=1}^{k} \omega_{j}^{2}$$

Here *σ_i_* is the standard deviation of probe set *i*, *p* is the number of probe sets, and *ω_j_* is the standard deviation explained by the selected principal component *j* (equal to the square root of the *j*’th eigenvalue). We determined the optimal number of components by the first local minimum of BIC in the scree plot (Figure 1).

#### Selection of representative probe sets in each component

Let **X** denote a *p* (number of probe sets) by *n* (number of samples) matrix with X*_ij_* denoting the expression level of probe set *i* in sample *j*. PCA determines the component score matrix **Y** and the weight matrix **u**, which are constrained as follows:

$$Y_{sj}=\sum_{i=1}^{p} u_{si}X_{ij}\text{ }\forall j\in[1,n]$$

$$\text{or }\mathbf{Y}_{s\times n}=\mathbf{u}_{s\times p}\mathbf{X}_{p\times n}$$

Here *s* is the index of principal component. Each component score *Y*_sj_ can be interpreted as a weighted average expression of all $X_{i\bullet}$ in the sample *j*, of which the weight vector $\vec{u}_{s}$ is equal to the *s*’th eigenvector of the covariance matrix of **X**.

To identify a subset of probe sets to represent each component *s*, $X_{i\bullet}$we determined the Pearson correlation coefficient between the component scores $Y_{s\bullet}$ and the expression levels $X_{i\bullet}$ and assessed the significance using student’s t-test. For each component *s*, we selected probe sets with a test p-value below 0.01. After the selection, each PC contains 42 to 211 representative probe sets.

### Identification of consistent components

Next we sought to combine the principal components from all five data sets, and to identify sets of similar components. However, each principal component *s* is represented by a different group of probe sets and corresponding weights (defined above, $\vec{u}_{s}$), so a direct comparison is not straightforward. We defined the following distance function *D* as a measure of the pair-wise dissimilarity between components *i* and *j*:

$$D_{ij}=(1-J_{ij})\times(1-C_{ij})$$

Where *J_ij_* is the Jaccard index (the ratio between size of the [intersection](http://en.wikipedia.org/wiki/Intersection_(set_theory)) and the size of the [union](http://en.wikipedia.org/wiki/Union_(set_theory)) of the representative probe sets of component *i* and *j*); *C_ij_* is the cosine correlation coefficient between the weights of the common representative probe sets of component *i* and *j*. In the DNBC study, *D_ij_* of components with less than 5 probes in common is set to be 1.

We used this distance function to perform average linkage hierarchical clustering on the selected principal components from all five data sets. We identified six distinct clusters, which we termed "consistent components". For each of the consistent components we converted the representative probe sets to gene symbols and chose the set of genes found in at least two member components as the representative genes for that consistent component **(Supplementary Data 1)**.

### Factor analysis and estimation of coefficients

We pooled all the genes from the six consistent components into a single list of 108 genes and retrieved the RMA expression profile of these genes from the five reference double-negative data sets. In case one gene was represented by multiple probe sets, we selected the probe set with the largest standard deviation to represent that gene. For each of the expression matrices retrieved, we computed the standard scores **Z***_i_* (difference between the expression values **X***_i_* and the population mean *µ_i_* divided by the standard deviation *σ_i_*) of every gene across all samples.

$$\mathbf{Z}_{i}=\frac{\mathbf{X}_{i}-\mu_{i}}{\sigma_{i}}$$

We then merged the five matrices of z-scores into one matrix **Z**, in which the columns correspond to all the samples from the five original data sets and the rows correspond to the 108 consistent component genes.

We performed factor analysis of the merged expression matrix **Z** with the number of factors set to six. Each factor contains a subset of 108 genes, and the loadings are given by the factor loading matrix **A**. Accordingly, the factor score matrix **F** was estimated by the least squares method on the following equation (11):

$$\mathop{\mathbf{F}}\limits^{\wedge} _{s\times p}=\mathbf{A}_{s\times n}^{T}\mathbf{R}_{n\times n}^{-1}\mathbf{Z}_{n\times p}$$

where **R** is the correlation matrix of **Z**, and **A***^T^* **R**^-1^ is the estimated regression coefficient matrix (weights) for the genes in the original expression matrix. Based on the estimated weights we defined two sets of metagenes: one used continuous weights equal to the regression coefficients; the other used simplified weights equal to the signs of the coefficients.

### Prediction of cancer outcomes based on Consistent Expression Indices (CEIs)

We collected six published breast cancer cohorts(1, 3-4, 12-15) and computed the CEI scores separately **(Supplementary Table 1)**. We pooled the CEI scores of the six cohorts and then grouped the samples into four subsets based on the treatment status and subtypes (DNBC vs ER positive HER2 negative, treated vs. untreated). For each of the subsets, we performed a classification based on the median of the six CEIs. The predictive power of the classification for five or ten-year clinical outcome was estimated separately by univariate Cox regression.

As for treatment response, we took four clinical trials for breast cancer(16-19) with expression profiles available and computed the CEI scores for each data set separately as described previously. We generated a Receiver Operating Curve (ROC) and used the Area Under the Curve (AUC) to estimate the predictive power of the CEIs, and we used Wilcoxon’s signed rank test to test the statistical significance.

**Comparison with Existing methods**

To compare our methods with other existing algorithms, we tested five supervised methods and two unsupervised methods.

For the supervised methods, we use MDA1 data set to derive the metagenes using Pearson’s correlation coefficient, diagonal linear discrimination analysis, student’s t-test, Wilcoxon’t rank sum test and nearest shrunken centroids, respectively(20-21). Except for PAM, which implements gene selection based on cross-validation embedded in the algorithm, we selected the predictive genes based on Bonferroni-corrected P-values of the corresponding statistics with an expected false positive rate of 0.05. Then we tested the selected genes in two independent data sets: MDA2 and EORTC for their prediction of pathological responses in the DNBC. The predictive power is evaluated using area under the receiver operating characteristic curve (ROC) and Wilcoxon’t rank sum test.

For unsupervised methods, we mean-centred the five DNBC data sets we used to derive the consistent principal components, pooled them into one data matrix. Then we subjected the pooled data matrix to sparse PCA (SPCA)(22) and independent component analysis (ICA)(23), respectively. We chose the top six components from each method to derive twelve metagenes. Then we validated the predictive power of these metagenes in five external validation cohorts received neoajuvant therapy of different regimens. Again the predictive power is evaluated using area under the receiver operating characteristic curve (ROC) and Wilcoxon’t rank sum test.

### Consistent Expression Indices in stage III ovarian cancer and early stage lung cancer

To further tested the consistent expression indices in other cancer types, we applied the same method to three ovarian cancer data sets(24-26) and four lung cancer data sets(27), respectively. For ovarian cancer, we derived three CEIs from the stage III ovarian cancer and tested these CEIs in another two independent data sets(28-29) for association to long-term clinical outcome as well as treatment responses. For lung cancer, we first performed a four-round cross validation in four data sets(27). In each round we derived CEIs from the early stage lung cancer samples in three of the data sets and then tested these CEIs in the one data set left out **(Supplementary Table 4)**. After the cross validation we derived six CEIs from all four lung cancer data sets and tested them in the fifth external data set(25) **(Supplementary Table 2, 4)**.

### Gene Ontology analysis

We analyzed the Gene Ontology for the genes selected in our analysis using the R package “GOstats”. We converted the genes to ENTREZ identifiers, and the significance of enrichment of the GO terms was tested against the hypergeometric distribution **(Supplementary Table 5)**.

### Reference

1. Wang Y*, et al.* (2005) Gene-expression profiles to predict distant metastasis of lymph-node-negative primary breast cancer. (Translated from eng) *Lancet* 365(9460):671-679 (in eng).

2. Doane AS*, et al.* (2006) An estrogen receptor-negative breast cancer subset characterized by a hormonally regulated transcriptional program and response to androgen. (Translated from eng) *Oncogene* 25(28):3994-4008 (in eng).

3. Loi S*, et al.* (2007) Definition of clinically distinct molecular subtypes in estrogen receptor-positive breast carcinomas through genomic grade. (Translated from eng) *J Clin Oncol* 25(10):1239-1246 (in eng).

4. Sotiriou C*, et al.* (2006) Gene expression profiling in breast cancer: understanding the molecular basis of histologic grade to improve prognosis. (Translated from eng) *J Natl Cancer Inst* 98(4):262-272 (in eng).

5. Lu X*, et al.* (2008) Predicting features of breast cancer with gene expression patterns. (Translated from eng) *Breast cancer research and treatment* 108(2):191-201 (in eng).

6. Matros E*, et al.* (2005) BRCA1 promoter methylation in sporadic breast tumors: relationship to gene expression profiles. (Translated from eng) *Breast cancer research and treatment* 91(2):179-186 (in eng).

7. Richardson AL*, et al.* (2006) X chromosomal abnormalities in basal-like human breast cancer. (Translated from eng) *Cancer Cell* 9(2):121-132 (in eng).

8. Irizarry RA*, et al.* (2003) Exploration, normalization, and summaries of high density oligonucleotide array probe level data. (Translated from eng) *Biostatistics* 4(2):249-264 (in eng).

9. Kaufman L & Rousseeuw P (1990) *Finding Groups in Data: An Introduction to Cluster Analysis* (John Wiley, New York).

10. Li C, Wong, Wing Hung (2003) DNA-Chip Analyzer (dChip). *The analysis of gene expression data: methods and software*, ed G Parmigiani EG, R Irizarry, SL Zeger (Springer).

11. Bartlett M (1937) The statistical conceptrion of metal factors. *British Journal of Psychology (Statistics Section)* 28:97-104.

12. Chin K*, et al.* (2006) Genomic and transcriptional aberrations linked to breast cancer pathophysiologies. (Translated from eng) *Cancer Cell* 10(6):529-541 (in eng).

13. Ivshina AV*, et al.* (2006) Genetic reclassification of histologic grade delineates new clinical subtypes of breast cancer. (Translated from eng) *Cancer Res* 66(21):10292-10301 (in eng).

14. Pawitan Y*, et al.* (2005) Gene expression profiling spares early breast cancer patients from adjuvant therapy: derived and validated in two population-based cohorts. (Translated from eng) *Breast Cancer Res* 7(6):R953-964 (in eng).

15. van de Vijver MJ*, et al.* (2002) A gene-expression signature as a predictor of survival in breast cancer. (Translated from eng) *N Engl J Med* 347(25):1999-2009 (in eng).

16. Farmer P*, et al.* (2009) A stroma-related gene signature predicts resistance to neoadjuvant chemotherapy in breast cancer. (Translated from eng) *Nat Med* 15(1):68-74 (in eng).

17. Hess KR*, et al.* (2006) Pharmacogenomic predictor of sensitivity to preoperative chemotherapy with paclitaxel and fluorouracil, doxorubicin, and cyclophosphamide in breast cancer. (Translated from eng) *J Clin Oncol* 24(26):4236-4244 (in eng).

18. Popovici VC, W. Gallas, BG. Pusztai, L et al (2009) Effect of training sample size and classification difficulty on the accuracy of genomic predictors. *Nature Biotechnol* NA(NA):NA.

19. Li Y*, et al.* (2010) Amplification of LAPTM4B and YWHAZ contributes to chemotherapy resistance and recurrence of breast cancer. (Translated from eng) *Nat Med* 16(2):214-218 (in eng).

20. Tibshirani R, Hastie T, Narasimhan B, & Chu G (2002) Diagnosis of multiple cancer types by shrunken centroids of gene expression. (Translated from eng) *Proc Natl Acad Sci U S A* 99(10):6567-6572 (in eng).

21. Hastie T, Tibshirani R, Friedman J, & Franklin J (2005) The elements of statistical learning: data mining, inference and prediction. *The Mathematical Intelligencer* 27(2):83-85.

22. Hui Zou TH, Robert Tibshirani. (2006) Sparse Principal Component Analysis *Journal of Computational and Graphical Statistics.* 2(15):22.

23. A. Hyvärinen JK, E. Oja (2001) *Independent Component Analysis* (Wiley, New York).

24. expO (2009) International Genomics Consortium (Phoenix).

25. Bild AH*, et al.* (2006) Oncogenic pathway signatures in human cancers as a guide to targeted therapies. (Translated from eng) *Nature* 439(7074):353-357 (in eng).

26. Tothill RW*, et al.* (2008) Novel molecular subtypes of serous and endometrioid ovarian cancer linked to clinical outcome. (Translated from eng) *Clin Cancer Res* 14(16):5198-5208 (in eng).

27. Shedden K*, et al.* (2008) Gene expression-based survival prediction in lung adenocarcinoma: a multi-site, blinded validation study. (Translated from eng) *Nat Med* 14(8):822-827 (in eng).

28. Ahmed AA*, et al.* (2007) The extracellular matrix protein TGFBI induces microtubule stabilization and sensitizes ovarian cancers to paclitaxel. (Translated from eng) *Cancer Cell* 12(6):514-527 (in eng).

29. Konstantinopoulos PA*, et al.* (2008) Carboplatin-induced gene expression changes in vitro are prognostic of survival in epithelial ovarian cancer. (Translated from eng) *BMC Med Genomics* 1:59 (in eng).
