# Supplementary figures and images for: Consistent metagenes from cancer expression profiles yield agent specific predictors of chemotherapy response
Source: BMC Bioinformatics. 2011 Jul 28;12:310. doi: 10.1186/1471-2105-12-310 (PMC3155975; doi:10.1186/1471-2105-12-310)

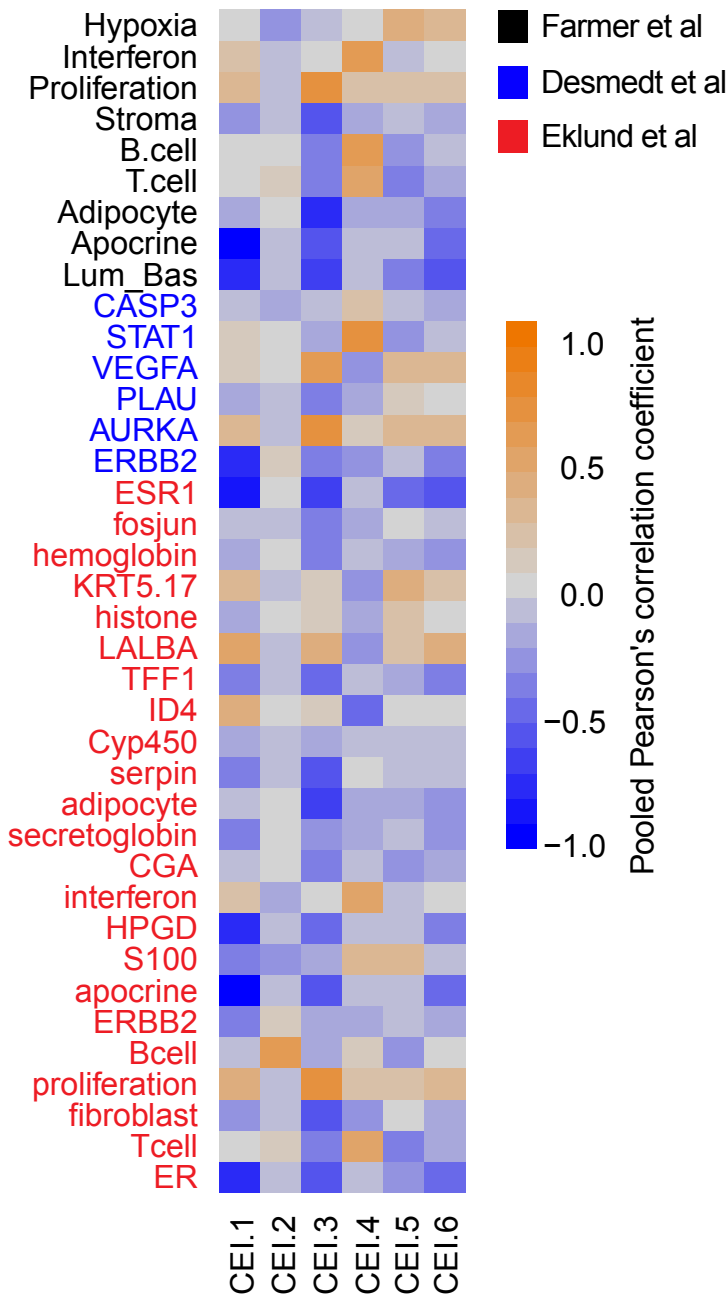

Supplement: Additional file 5 — Correlation between DNBC-derived CEIs and known metagenes. Colorgram showing the pooled Pearson correlation coefficients between DNBC-derived CEIs and known metagenes. [file 1471-2105-12-310-S5.PDF]

**a.**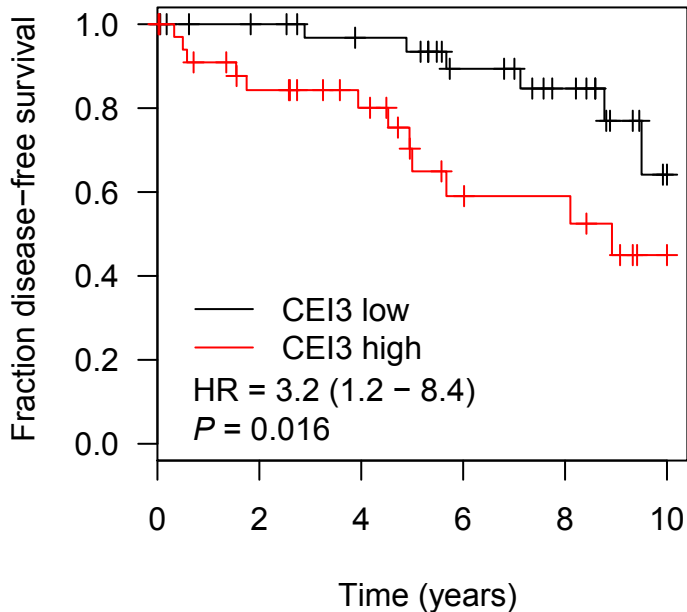

number at risk

|    |    |    |    |    |   |
|----|----|----|----|----|---|
| 37 | 33 | 29 | 21 | 15 | 3 |
| 35 | 25 | 19 | 10 | 9  | 3 |

**b.**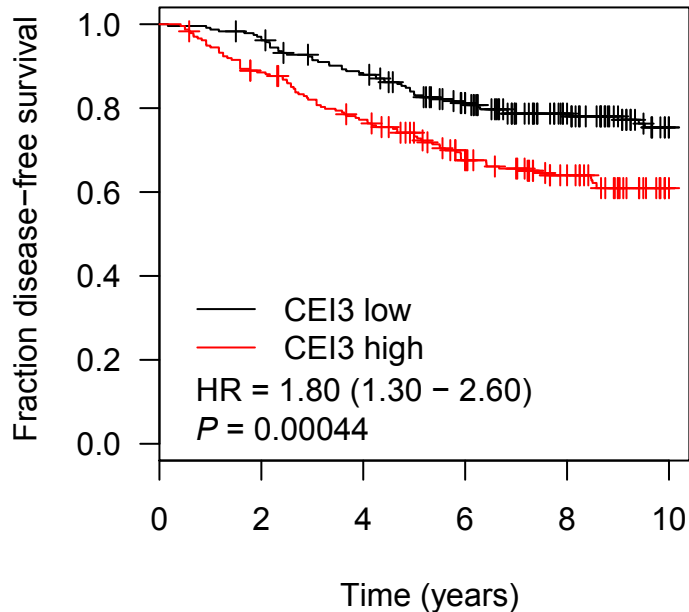

Number at risk

|     |     |     |     |     |    |
|-----|-----|-----|-----|-----|----|
| 235 | 227 | 203 | 170 | 122 | 76 |
| 236 | 206 | 177 | 140 | 114 | 76 |

Supplement: Additional file 7 — DNBC-derived CEI3 predict clinical outcome of in ER-positive HER2-negative breast cancer. (a) ER-positive HER2-negative samples which received endocrine or radio-therapy from the EMC, JBI1, GIS, KUH, UCSF and NKI cohorts; (b) ER-positive HER2-negative samples which received no systematic therapy. [file 1471-2105-12-310-S7.PDF]
