# Supplementary material for: Consistent metagenes from cancer expression profiles yield agent specific predictors of chemotherapy response
Source: BMC Bioinformatics. 2011 Jul 28;12:310. doi: 10.1186/1471-2105-12-310 (PMC3155975; doi:10.1186/1471-2105-12-310)
Supplement: Additional file 6 — AUCs for prediction of pathological response in five DNBC cohorts which received neoadjuvant chemotherapy of different regimens using various unsupervised methods. (a) CEIs derived from consistent principal components; (b) Components derived using independent component analysis; (c) Components derived using sparse principal component analysis. The pooled correlation coefficients were estimated from seven breast cancer data sets based on a meta-analysis. [file 1471-2105-12-310-S6.DOC]

**Supplementary Table 5.** Each metagene was evaluated as a univariate predictor of pathological complete response or residual disease using the area under the ROC curve (AUC). Chemotherapy regimens are indicated: A, doxorubicin; C, cyclophosphamide; E, epirubicin; F, 5-fluorouracil; P, either cisplatin or carboplatin; T, either paclitaxel or docetaxel. The CEIs were derived from four independent DNBC cohorts not shown in this table. * *P* < 0.05; ** *P* < 0.01. R: AUC is estimated based on association to residual disease (RD).

**(a)** CEIs derived using consistent principal components.

|  | | | | AUC | | | | | |
| --- | --- | --- | --- | --- | --- | --- | --- | --- | --- |
| cohort | regimen | patients | responders | CEI1 | CEI2 | CEI3 | CEI4 | CEI5 | CEI6 |
| EORTC | FEC | 37 | 16 | 0.73^R^* | 0.57 | 0.51^R^ | 0.61 | 0.56 | 0.54 |
| MDA1 | TFAC | 27 | 13 | 0.78 ** | 0.62 | 0.77** | 0.61^R^ | 0.53 | 0.61 |
| MDA/MAQC | TFAC | 30 | 9 | 0.77* | 0.66 | 0.78* | 0.62^R^ | 0.58 | 0.54 |
| DFCI2 | P | 24 | 4 | 0.73 | 0.72^R^ | 0.50 | 0.52^R^ | 0.52^R^ | 0.57^R^ |
| JBI2 | E | 43 | 4 | 0.85^R^* | 0.73^R^ | 0.53 | 0.88** | 0.58^R^ | 0.72 |

**(b)** Components derived using independent component analysis

|  | | | | AUC | | | | | |
| --- | --- | --- | --- | --- | --- | --- | --- | --- | --- |
| cohort | regimen | patients | responders | ICA1 | ICA2 | ICA3 | ICA4 | ICA5 | ICA6 |
| EORTC | FEC | 37 | 16 | 0.51 | 0.51^R^ | 0.64^R^ | 0.67 | 0.52 | 0.56 |
| MDA1 | TFAC | 27 | 13 | 0.56^R^ | 0.60^R^ | 0.72* | 0.52^R^ | 0.72* | 0.75^R^* |
| MDA/MAQC | TFAC | 30 | 9 | 0.55 | 0.58 | 0.74^R^* | 0.59 | 0.57^R^ | 0.72^R^ |
| DFCI2 | P | 24 | 4 | 0.72 | 0.58 | 0.52 | 0.67^R^ | 0.78^R^ | 0.54^R^ |
| JBI2 | E | 43 | 4 | 0.53 | 0.72 | 0.70 | 0.62 | 0.53 | 0.78 |

**(c)** Components derived using sparse principal component analysis.

|  | | | | AUC | | | | | |
| --- | --- | --- | --- | --- | --- | --- | --- | --- | --- |
| cohort | regimen | patients | responders | SPCA1 | SPCA2 | SPCA3 | SPCA4 | SPCA5 | SPCA6 |
| EORTC | FEC | 37 | 16 | 0.60 | 0.67 | 0.65^R^ | 0.62^R^ | 0.54 | 0.66^R^ |
| MDA1 | TFAC | 27 | 13 | 0.69^R^ | 0.57 | 0.72* | 0.59^R^ | 0.51 | 0.76** |
| MDA/MAQC | TFAC | 30 | 9 | 0.57 | 0.64 | 0.53^R^ | 0.52^R^ | 0.62^R^ | 0.57 |
| DFCI2 | P | 24 | 4 | 0.61 | 0.69^R^ | 0.70 | 0.64 | 0.66 | 0.58 |
| JBI2 | E | 43 | 4 | 0.85* | 0.57^R^ | 0.61^R^ | 0.62 | 0.73^R^ | 0.72^R^ |
