# Supplementary material for: Consistent metagenes from cancer expression profiles yield agent specific predictors of chemotherapy response
Source: BMC Bioinformatics. 2011 Jul 28;12:310. doi: 10.1186/1471-2105-12-310 (PMC3155975; doi:10.1186/1471-2105-12-310)
Supplement: Additional file 8 — Validation of the association between CEIs and clinical outcomes in ovarian cancers and lung cancers. (a) Hazard ratios based on 5-year follow-up of three ovarian cancer-derived CEIs in the validation cohort (DU) based on univariate and multivariate Cox regression; (b) Summary cross-validation of CEIs derived from three early-stage lung cancer data sets and validated in the fourth for the association to clinical outcomes; (c) hazard ratios based on 5-year follow-up of seven lung cancer-derived CEIs in the validation cohort (DU) based on univariate and multivariate Cox regression. [file 1471-2105-12-310-S8.DOC]

**Supplementary Table 4** Validation of the association between CEIs and clinical outcome in ovarian cancers and lung cancers.

**(a)** Hazard ratios based on 5-year follow-up of three ovarian cancer-derived CEIs in the validation cohort (DU) based on univariate and multivariate Cox regression

| **Ovarian cancer-derived** | **Univariate, BIDMC** | **Log-rank P** | **Multivariate, BIDMC** | **Log-rank P** |
| --- | --- | --- | --- | --- |
| **CEI1** | 8.36 (1.63 – 43.0) | 0.011 | 10.45 (1.61 – 68.03) | 0.014 |
| **CEI2** | 1.67 (0.60 – 4.67) | 0.33 | 2.11 (0.81 – 5.51) | 0.130 |
| **CEI3** | 2.15 (0.69 – 6.71) | 0.19 | 0.93 (0.22 – 3.85) | 0.920 |

**(b)** Summary cross-validation of CEIs derived from three early-stage lung cancer data sets and validated in the fourth for the association to clinical outcomes.

| **Lung cancer data used to derive CEIs** | **CAN/DF**  **HLM**  **UM** | **MSK**  **HLM**  **UM** | **MSK**  **CAN/DF**  **UM** | **MSK**  **CAN/DF**  **HLM** | | |
| --- | --- | --- | --- | --- | --- | --- |
| **Validation data (univariate)** | **MSK** | **CAN/DF** | **HLM** | **UM** |  | |
| **CEI1** | 0.81 (0.09 – 7.46)  P = 0.85 | 0.83 (0.17 – 4.04)  P = 0.81 | 0.15 (0.03 – 0.83)  P = 0.027 | 1.10 (0.46 – 2.62)  P = 0.83 | |  |
| **CEI2** | 0.99 (0.10 – 10.31)  P = 1.00 | 0.58 (0.10 – 3.13)  P = 0.52 | 4.00 (1.34 –11.91)  P = 0.010 | 1.22 (0.43 – 3.43)  P = 0.71 | |  |
| **CEI3** | 0.22 (0.05 – 0.95)  P = 0.038 | 1.47 (0.32 – 6.73)  P = 0.62 | 0.65 (0.17 – 2.51)  P = 0.53 | 2.65 (1.21 – 5.79)  P = 0.013 | |  |
| **CEI4** | 0.62 (0.08 – 4.92)  P = 0.65 | 0.40 (0.05 – 2.98)  P = 0.37 | 2.48 (0.70 – 8.70)  P = 0.15 | 1.04 (0.37 – 2.95)  P = 0.93 | |  |
| **CEI5** | 0.24 (0.03 – 1.91)  P = 0.18 | 0.35 (0.03 – 3.74)  P = 0.38 | – | 0.62 (0.19 – 2.02)  P = 0.43 | |  |

**(c)** hazard ratios based on 5-year follow-up of seven lung cancer-derived CEIs in the validation cohort (DU) based on univariate and multivariate Cox regression.

| **Lung cancer-derived** | **Univariate, DU** | **Log-rank P** | **Multivariate, DU** | **Log-rank P** |
| --- | --- | --- | --- | --- |
| **CEI1** | 7.73 (1.15 – 52.16) | 0.034 | 50.36 (2.65 – 955.98) | 0.0091 |
| **CEI2** | 3.64 (0.65 – 20.44) | 0.139 | 2.52 (0.11 – 55.42) | 0.560 |
| **CEI3** | 0.20 (0.04 – 1.13) | 0.064 | 0.02 (0 – 0.25) | 0.0026 |
| **CEI4** | 1.85 (0.28 – 12.21) | 0.521 | 0.08 (0– 1.51) | 0.093 |
| **CEI5** | 1.66 (0.38 – 7.25) | 0.499 | 1.44 (0.16 – 13.31) | 0.750 |
| **CEI6** | 2.47 (0.43 – 14.14) | 0.304 | 1.91 (0.16 – 22.53) | 0.610 |
| **CEI7** | 0.22 (0.01 – 5.75) | 0.360 | 0.01 (0 – 0.78) | 0.038 |
